# Comparative Evaluation of the Antioxidant and Anti-Alzheimer’s Disease Potential of Coumestrol and Puerarol Isolated from *Pueraria lobata* Using Molecular Modeling Studies

**DOI:** 10.3390/molecules23040785

**Published:** 2018-03-28

**Authors:** Prashamsa Koirala, Su Hui Seong, Hyun Ah Jung, Jae Sue Choi

**Affiliations:** 1 Department of Food and Life Science, Pukyong National University, Busan 48513, Korea; prashamsakoirala20@gmail.com; 2 Department of Food Science and Human Nutrition, Chonbuk National University, Jeonju 54896, Korea; seongsuhui@naver.com

**Keywords:** cholinesterase, BACE1, molecular docking, kinetics, Alzheimer’s disease

## Abstract

The current study assesses the antioxidant effects of two similar isoflavonoids isolated from *Pueraria lobata*, coumestrol and puerarol, along with the cholinergic and amyloid-cascade pathways to mitigate Alzheimer’s disease (AD). Antioxidant activity was evaluated via 1,1-diphenyl-2-picryhydrazyl (DPPH) and peroxynitrite (ONOO^−^) scavenging ability further screened via ONOO^−^-mediated nitrotyrosine. Similarly, acetyl- and butyrylcholinesterase (AChE/BChE) and β-site amyloid precursor protein cleaving enzyme 1 (BACE1) inhibitory activities were assessed together with docking and kinetic studies. Considering DPPH and ONOO^−^ scavenging activity, coumestrol (EC_50_ values of 53.98 and 1.17 µM) was found to be more potent than puerarol (EC_50_ values of 82.55 and 6.99 µM) followed by dose dependent inhibition of ONOO^−^-mediated nitrotyrosine. Coumestrol showed pronounced AChE and BChE activity with IC_50_ values of 42.33 and 24.64 µM, respectively, acting as a dual cholinesterase (ChE) inhibitor. Despite having weak ChE inhibitory activity, puerarol showed potent BACE1 inhibition (28.17 µM). Kinetic studies of coumestrol showed AChE and BChE inhibition in a competitive and mixed fashion, whereas puerarol showed mixed inhibition for BACE1. In addition, docking simulations demonstrated high affinity and tight binding capacity towards the active site of the enzymes. In summary, we undertook a comparative study of two similar isoflavonoids differing only by a single aliphatic side chain and demonstrated that antioxidant agents coumestrol and puerarol are promising, potentially complementary therapeutics for AD.

## 1. Introduction

Among elderly patients, the most prevalent disease of the modern age is Alzheimer’s disease (AD). It is comprised of amyloid plaques consisting of β-amyloid (Aβ) peptides and neurofibrillary tangles of hyperphosphorylated tau protein which results in neuronal cell loss with dementia like symptom [1]. Of the two most prevalent current hypotheses, the amyloid cascade and cholinergic hypotheses, the amyloid cascade hypothesis illustrates Aβ as the pathological causative agent for dementia [2]. β-Site amyloid precursor protein (APP) cleaving enzyme 1 (BACE1) is the β-secretase enzyme required for the production of neurotoxic Aβ together with γ-secretase. The formation of Aβ is a sequential proteolytic process beginning with cleavage of APP by the β-secretase enzyme. Next, the remaining C99 is further cleaved by γ-secretase to release Aβ. Various studies have thus explored the production of BACE1 inhibitors to slow down the formation of Aβ [3]. Similarly, the cholinergic hypothesis is widely accepted and has been the focus of many AD investigations. The cholinergic hypothesis of AD states that cholinergic dysfunction may not cause cognitive impairment directly, but instead interferes with attentional processing, thereby causing dementia [4]. The recovery of cholinergic transmitter levels via acetyl- and butyrylcholinesterase (AChE/BChE) inhibitors has been proposed as the most effective target for AD treatment [5].

Oxidation is responsible for the pathogenesis of various age-related degenerative diseases such as cancer, diabetes, macular degeneration, AD, and Parkinson’s disease, as reactive pro-oxidant species can damage proteins, lipids, carbohydrates, and nucleic acids over time [6]. In addition, various studies have suggested that oxidation induces and activates multiple cell signalling pathways that contribute to lesion formation of toxic substances, finally potentiating AD. In fact, oxidative stress resulting from an increase in reactive oxygen species (ROS) and reactive nitrogen species (RNS) has also been thought to play a malicious role in AD progression [7]. It is thus a salient feature of neurodegeneration linked to the aging process that exacerbates AD [8]. However, there are doubts as to whether antioxidants play a beneficial role in mitigating AD, while there is ample evidence suggesting the beneficial effects of antioxidants [9]. As a result, our study aims to provide additional evidence regarding antioxidant use.

Despite the availability of cholinesterase (ChE) inhibitors like donepezil, galantamine, and rivastigmine for AD, adverse effects still outweigh the benefit of commercial drugs over natural products. The most common side effects related to cholinergic stimulation in the brain and peripheral tissues include, gastrointestinal, cardiorespiratory, extrapyramidal, genitourinary, and musculoskeletal symptoms, as well as sleep disturbance [10]. Different generations of BACE1 inhibitors are also available, such as E2609 and verubecestat; however, side effects including liver toxicity, low oral bioavailability, and low efficacy have limited their use [11]. The significance of natural products in healthcare was supported by a report that 80% of the global population still relies on plant derived medicines to address their health care needs. It was also reported that 50% of all drugs in clinical use are natural products, and 74% of the most important drugs consist of plant-derived active ingredients [12]. Thus, our interest in plant-based drugs has guided us to study the anti-Alzheimer’s disease potential of two major *Pueraria lobata*-derived antioxidant compounds.

Coumestrol is a phytoestrogen occurring in plants in the coumestan family of compounds; it shares a common structure with isoflavones and estradiol and exhibits estrogenic and antiestrogenic activity based on estrogen levels in the body [13]. Research on such potential phytoestrogens has been ongoing since ancient times. It was first isolated from ladino clover in 1956; it is widely distributed in plants like clover, soya beans, peas, and strawberries [14]. In a successive research paper, Bickoff et al. [15] elaborated on 13 years of research on coumestrol, which highlighted its pharmacological importance. Coumestrol is able to pass through cell membranes due to its low molecular weight and stable structure and was reported to exhibit a neuroprotective effect via cerebral ischemia prevention [16]. Foodstuffs containing coumestrol exert beneficial effects in cancer, menopause, osteoporosis, atherosclerosis, and cardiovascular disease [17]. In addition, it has been reported to exhibit anti-aging [18], neuroprotective [19], anti-adipogenic [20], and depigmenting activity [21]. With such diverse potential, further investigation of its neuroprotective properties is necessary. On the other hand, the coumestan derivative puerarol which has a common structure with coumestrol is a compound with high potential that has been overlooked. Thus, our study aimed to elucidate the nature of puerarol and to shed more light on such potentially interesting compounds. Another interesting fact about these two compounds is they share a common structure differing only by an aliphatic side chain in puerarol.

**Figure 1:**
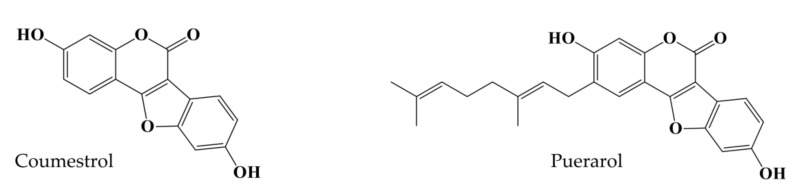
Structure of coumestrol and puerarol.

Our previous work on *P. lobata*-derived compounds illustrated obvious anti-AD [22], anti-diabetic [23], and hepatoprotective potential [24]. In the current study, we sought to explore the potential of two similar isoflavonoids in the prevention of AD by assimilation of two approaches: antioxidant therapy and amyloid cascade/cholinergic pathways. These approaches would enable protection against AD via a multifaceted mechanism while also illuminating structure-activity relationships. Furthermore, the antioxidant potential of coumestrol and puerarol was visualized by western blot analysis for inhibition of peroxynitrite (ONOO^−^)-mediated nitrotyrosine formation, while chemical kinetics and molecular docking studies cleared the mechanism of AD (Figure 1).

## 2. Results

### 2.1. DPPH and ONOO^−^ Scavenging Potentials of Coumestrol and Puerarol

As illustrated in Table 1, coumestrol had potent 2,2-diphenyl-1-picrylhydrazyl (DPPH) scavenging activity with an EC_50_ value of 53.98 ± 1.00 µM, while puerarol showed a reasonable EC_50_ of 82.55 ± 1.33 µM. *L*-Ascorbic acid was used as a positive control with an EC_50_ value of 17.37 ± 0.32 µM. In terms of ONOO^−^ scavenging activity, coumestrol was very potent with an EC_50_ value of 1.17 ± 0.11 µM that was five times more potent than puerarol (6.99 ± 0.30 µM) as well as the positive control *L*-penicillamine (6.9 ± 0.08 µM).

### 2.2. ONOO^−^-Mediated Western Blot Analysis of Coumestrol and Puerarol

**Figure 2:**
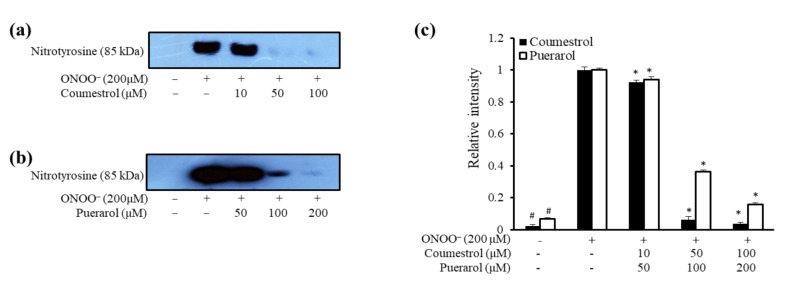
Dose-depended inhibition of ONOO^−^-mediated albumin nitration by coumestrol and puerarol. Mixtures of test samples, bovine serum albumin (BSA), and ONOO^−^ were incubated with shaking at 37 °C for 30 min. The reactant was resolved in 10% polyacrylamide gel via electrophoresis. (**a**) Coumestrol; and (**b**) puerarol, were used at the indicated concentrations; (**c**) Quantification of band intensity was calculated using CS Analyzer 3.00 (ATTO Corp., Tokyo, Japan). ^#^ *p* < 0.05 indicates a significant difference from the untreated normal group, * *p* < 0.05 indicate significant differences from the ONOO^−^ treated control.

The western blot is an analytical technique used to detect nitrated bovine serum albumin (BSA) in a mixture of BSA, ONOO^−^, and test samples. Figure 2 shows the blot analysis of coumestrol and puerarol as a pictorial view. Blot analysis of coumestrol demonstrated a dose dependent decrease in the blot intensity from lower concentration (10 µM) to higher concentration (100 µM). A slimmer blot at 100 µM indicates the potential of coumestrol to decrease ONOO**^−^** formation. Double the concentration for puerarol also showed a dose dependent decrease in the blot intensity (50–200 µM).

### 2.3. Anti-AD Potential of Coumestrol and Puerarol

Coumestrol showed potent AChE, BChE, and BACE1 inhibitory activities with IC_50_ values of 42.33 ± 1.29, 24.64 ± 2.28, and 51.04 ± 1.86 µM, respectively, compared to the positive controls berberine and quercetin, as shown in Table 2. Coumestrol has also been shown to act as a dual ChE inhibitor. Puerarol showed moderate AChE inhibitory activity with an IC_50_ value of 144.80 ± 2.46 µM, although BChE inhibitory activity was very weak. However, it had potent BACE1 inhibitory activity with an IC_50_ value of 28.17 ± 2.48 µM, comparable to the positive control quercetin (21.28 ± 1.42 µM). Thus, we performed enzyme kinetic analysis and molecular docking studies with coumestrol for ChEs and with puerarol for BACE1.

### 2.4. Enzyme Kinetic Analysis of Coumestrol and Puerarol for ChEs and BACE1

**Figure 3:**
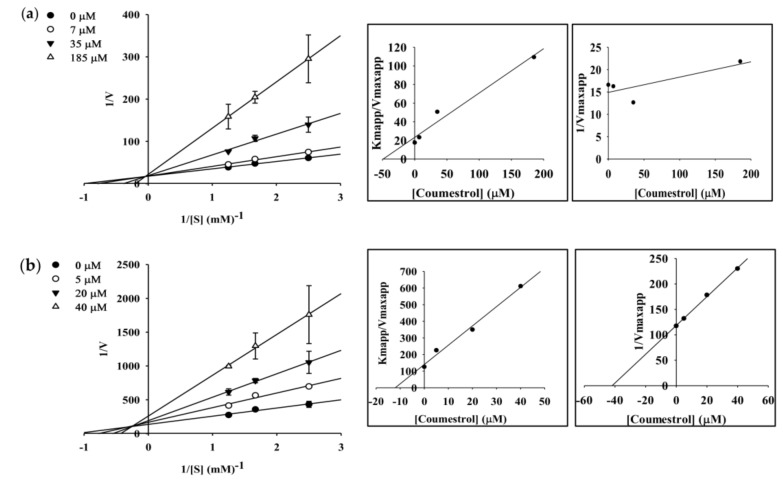
Lineweaver-Burk plots and secondary plots for AChE (**a**) and BChE (**b**) inhibition of coumestrol.

**Figure 4:**
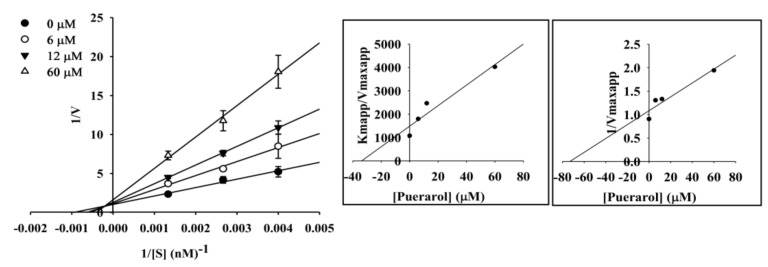
Lineweaver-Burk plots and secondary plots for BACE1 inhibition of puerarol.

To elucidate the mechanisms of ChE inhibition, kinetic studies of enzyme activity were performed. The slope or intercept in Lineweaver-Burk plots were drawn in SigmaPlot 12.0 as a function of inhibitor concentration, and the kinetic parameters were determined. As shown in Table 2, coumestrol showed a competitive mode of inhibition for AChE and a mixed type for BChE with inhibition constant (*K_i_*) values of 48.91 µM for AChE and 12.07 (*K_ic_*) and 41.87 (*K_iu_*) µM for BChE (Figure 3 and Table 2). The same *y* (*V_max_*) intercept in the Lineweaver-Burk plot demonstrated competitive inhibition, and the different *x*-(*K_m_*) and *y*-axis showed mixed type inhibition. For puerarol, BACE1 inhibition kinetic studies of enzyme activity were performed, which showed a mixed type of inhibition with *K_ic_* and *K_iu_* values of 33.8 and 73.19 µM, respectively (Figure 4 and Table 2).

### 2.5. Molecular Docking Simulation Studies of Coumestrol on ChEs

**Figure 5:**
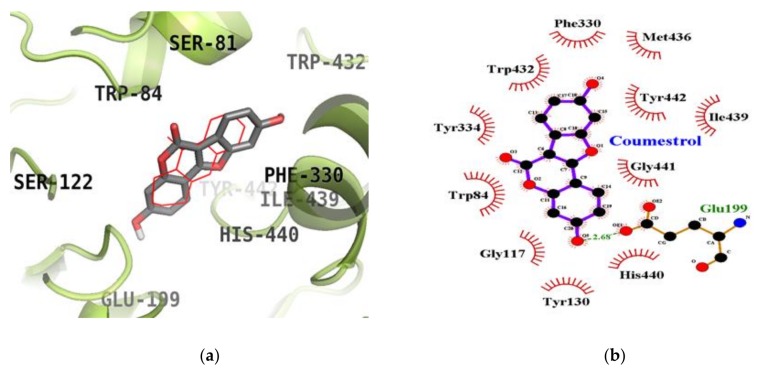
Inhibition mode of coumestrol for the AChE (**a**) catalytic site with tacrine (red line). 2D ligand interaction diagram of AChE inhibition by coumestrol (**b**). Dashed lines indicate H-bonds. Carbons are in black, nitrogens in blue, and oxygens in red.

**Figure 6:**
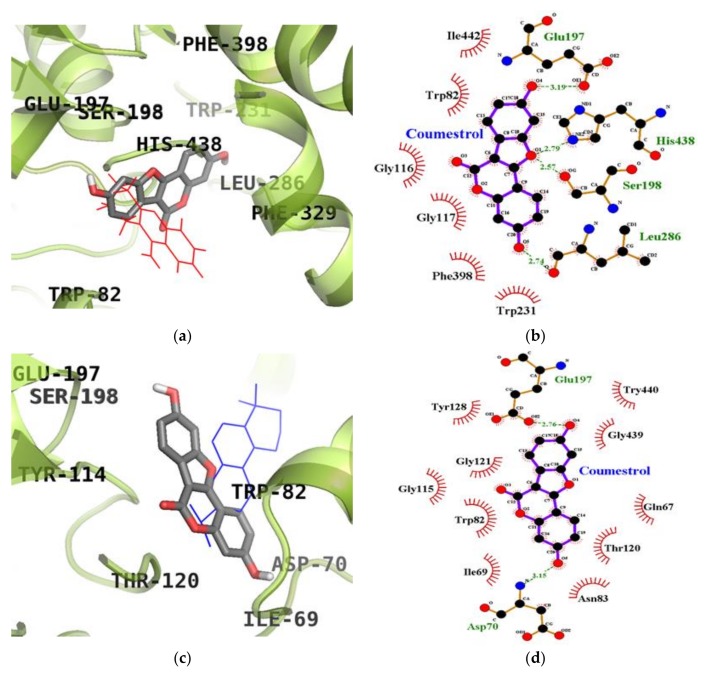
Inhibition mode of coumestrol for the BChE catalytic site with tacrine (red line) (**a**) and allosteric site with cryptotanshinone (blue line) (**c**). 2D ligand interaction diagram of BChE catalytic (**b**) and allosteric (**d**) inhibition by coumestrol. Dashed lines indicate H-bonds. Carbons are in black, nitrogens in blue, and oxygens in red.

We next used molecular docking studies to obtain accurate predictions of protein-ligand interaction geometries for coumestrol. Docking scores for the selected compounds with interacting residues, as well as the number of hydrogen bonds formed between interacting residues and hydrophobic interacting residues are shown in Table 3. We found that the activity of coumestrol against ChEs correlated with the binding energy and the number of hydrogen bonds formed in the active site of AChE and BChE. The top binding energy of coumestrol towards AChE (PDB ID: 1ACJ) was −8.63 kcal/mol. As shown in Figure 5a,b, coumestrol bound to the Glu199 residue (located in the quaternary ammonium binding site) of AChE through the formation of a hydrogen bond. Moreover, important catalytic residue, His440, was found to form hydrophobic interactions. The top binding site of coumestrol against BChE (PDB ID: 4BDS) was placed in an allosteric pocket with a binding energy of −8.67 kcal/mol. In this configuration, coumestrol strongly bound to Asp70, an important peripheral anionic site (PAS) residue of BChE, and Glu197 residues via two hydrogen bonds. In addition to these hydrogen bonds, Gln67, Ile69, Trp82, Asn83, Gly115, Thr120, Gly121, Tyr128, Gly439, and Try440 residues interacted with coumestrol via hydrophobic interactions. Moreover, coumestrol bound to the catalytic pocket of BChE with a binding energy of −8.28 kcal/mol. Coumestrol bound to Glu197, Ser198, and Leu286 residues as well as the major catalytic residue, His438, via hydrogen bonds (Figure 6a,b) at the catalytic site of BChE.

### 2.6. Molecular Docking Simulation Studies of Puerarol on BACE1

**Figure 7:**
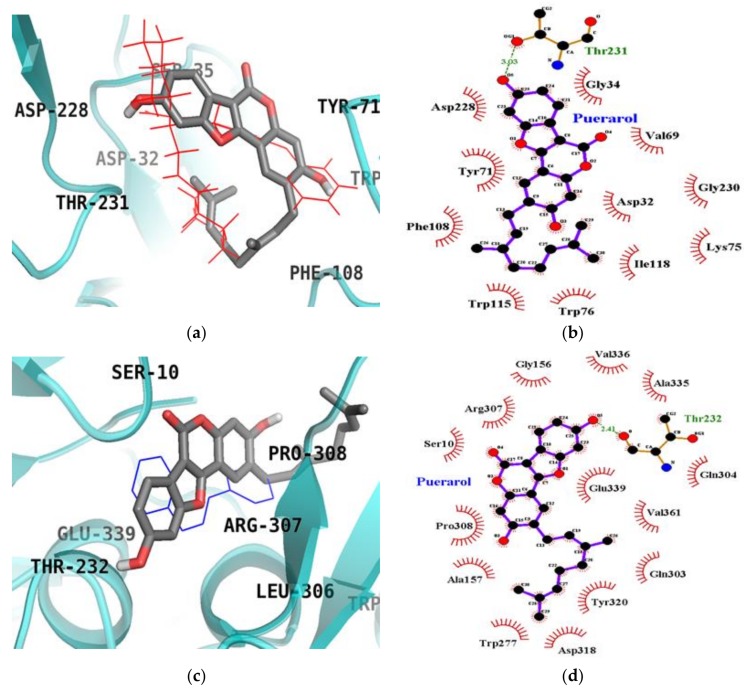
Inhibition mode of puerarol for the BACE1 catalytic site (**a**) with QUD (red line) and allosteric site (**c**) with TMF (blue line). 2D ligand interaction diagram of BACE1 catalytic (**b**) and allosteric (**d**) inhibition by puerarol. Dashed lines indicate H-bonds. Carbons are in black, nitrogens in blue, and oxygens in red.

The top binding configuration of puerarol against BACE1 (PDB ID: 2WJO) was similar to the catalytic ligand, 2-amino-3-{(1*R*)-1-cyclohexyl-2-[(cyclohexylcarbonyl) amino] ethyl}-6-phenoxy quinazolin-3-ium (QUD), with a binding energy of −8.80 kcal/mol. As shown in Figure 7a,b, the Thr231 residue was identified as an H-bond interacting residue in the catalytic inhibition mode of puerarol. In this complex, important catalytic aspartyl residues (Asp32 and Asp228) participated in hydrophobic interactions (Table 4). Like the enzyme kinetic results, puerarol also bound to an allosteric site of BACE1 with a binding energy of −8.03 kcal/mol. As shown in Figure 7c,d, puerarol interacted with allosteric residue Thr232 of BACE1 via a hydrogen bond in addition to binding with Ser10, Gly156, Ala157, Trp277, Gln303, Gln304, Arg307, Pro308, Asp318, Tyr320, Ala335, Val336, Gln339 and Val361 residues via hydrophobic interactions.

## 3. Discussion

A myriad of approaches to AD treatment are available. ROS generation is thought to be the most common target, and several antioxidant therapies have been used to address it. Oxidative and nitrosative stress are associated with the formation and accumulation of Aβ and BACE1, which several studies have affirmed [25,26,27]. Nitric oxide and superoxide anion react to yield ONOO^−^, which has been implicated in Aβ aggregation observed in the brain of AD patients [28]. Thus, with the aim to implement both antioxidant and neuroprotective activity to prevent AD, the combined effects of the two compounds were assessed. A study by Booth et al. [29] reported the inactivity of coumestrol regarding DPPH scavenging, which is contrary to our findings that coumestan-type compounds have definite scavenging potential through hydrogen/electron donation via hydroxyl groups. In addition, recent data indicated that the potent radical scavenging ability of soya beans is attributed to a type of phytoalexin, coumestrol, which has inherent antimicrobial and antioxidant activity, providing additional support for our postulate [30]. Likewise, a coumestan wedelolactone with a similar ring structure to coumestrol had been reported to exhibit potent DPPH scavenging activity with an IC_50_ value of 7 µM justifying the potential of coumestrol [31]. Numerous researchers have speculated that intraneuronal Aβ toxicity (Aβ40 and Aβ42) and secretases (α-secretase and β-secretase) might play important roles in the release of reactive oxygen intermediates and NO^•^. Based on the interconnectedness of reactive oxygen intermediates NO^•^, and AD, and how an antioxidant mechanism can ultimately alleviate AD, our study evaluated the peroxynitrite scavenging potential through inhibition of ONOO^−^-mediated nitrotyrosine formation via western blot analysis.

From a structural point of view, both isoflavonoids are identical with the exception of an aliphatic side chain. Both isoflavonoids potently reduced protein nitration as demonstrated by western blot, thereby inhibiting ChEs and BACE1, suggesting that the benzofuran ring is key to the exhibited anti-oxidant activity. However, the aliphatic side chain in puerarol seemed to decrease activity due to steric hindrance. Coumestrol, which is devoid of the side chain, showed potent antioxidant and anti-AD activities, whereas puerarol had comparatively weaker activity due to the presence of the aliphatic side chain. Interestingly, puerarol showed potent BACE1 activity despite having the side chain; further investigation is needed to explain this observation.

We analyzed the ChE and BACE1 inhibitory properties of coumestrol and puerarol via in vitro inhibitory assays supported by kinetics and computer aided molecular binding analysis. Coumestrol has drawn special attention from researchers, being a potent phytoestrogen. Coumestrol also exhibited potent ChEs inhibitory activity in our current study, which might be correlated with the hypothesis of Castro et al. [16], that it is a selective estrogen receptor modulator with inherent neuroprotective activity. Coumestrol shares a similar structure with isoflavones like genistein and diadzin that are known to exhibit potent AChE inhibitory activity [32]. In addition, a similar study by Ahmad et al. [33] revealed the ability of soybean and Temphe isoflavones including daidzein and genistein along with their glycosides to inhibit BACE1. Coumestrol contains a phenolic group similar to genistein with a similar electrostatic and configurational nature responsible for their activity. The current study identified coumestrol as an active dual inhibitor against ChEs and BACE1. Unlike conventional inhibitors, coumestrol exerts inhibitory activity against both hypothesized mechanisms of AD, providing a greater beneficial advantage. Despite weak BChE inhibitory activity, the BACE1 activity of these compounds is very interesting, which suggests the need for further evaluation.

The cumulative results suggestive of ChE as well as BACE1 inhibitory potential inspired us to further determine the type and mode of interaction via enzyme kinetic study. Coumesterol showed potent ChE activity, in agreement with a low *K_i_* value determined by kinetic parameters. Puerarol showed potent BACE1 activity, in agreement with a low *K_i_* value demonstrated by the secondary plot showing mixed type inhibition. Competitive inhibitors bind to catalytic sites of an enzyme and decrease the amount of binding of a substrate or ligand to the enzyme. Coumestrol displayed competitive inhibition against AChE and mixed inhibition against BChE, indicating that it may bind to the enzyme-substrate complex or interact with a specific catalytic or allosteric site of the enzyme. As a mixed inhibitor, coumestrol was able to bind either free BChE or the BChE-substrate complex, while puerarol bound to free BACE1 or the BACE1-substrate complex. In mixed inhibition, at sufficiently high substrate concentrations, the enzyme is exclusively present in the form of enzyme-substrate complex, and an inhibitor acts primarily as an uncompetitive inhibitor, which attempts to bind to the complex. Under this condition, a higher concentration of inhibitor is required to effectively inhibit the enzyme [34].

In the present study, molecular docking enabled us to investigate the activity, orientation, and interaction and binding energies. Beyond the goal of using molecular docking to predict binding affinities, modeling also allowed us to confirm the ChE and BACE1 inhibitory activity of both compounds and the inhibition mode through chemical kinetics. The docked ligand molecules were selected based on docking energy and good interaction with active site residues. In our subsequent experiments, 3D docking of the potent ChEs and BACE1 inhibitor, coumestrol, exhibited a minimum docking score for ChEs. A lower docking score indicates a greater binding capacity for the ligand. Hence, the docking scores and binding interactions of coumestrol were significantly associated with its ability to inhibit ChE activity. Regarding the apoptotic potential of coumestrol, a previous report determined the structural stability of coumestrol docked to the estrogen receptor (α and β) by analyzing H-bonds and the interaction energy, suggesting that coumestrol upon interaction with estrogen receptor-α led to a strong substrate binding affinity [35]. However, in our study, coumestrol was able to bind to the catalytic and allosteric sites of BChE. Coumestrol formed a hydrogen bond with His438, a major catalytic residue of BChE, and with the peripheral anionic site (Asp70 residue) of BChE. On the other hand, coumestrol interacted with His440, an important catalytic residue of AChE, specifically through hydrophobic interactions. Thus, coumestrol exhibited BChE inhibitory activity against the target protein in terms of binding efficiency. In particular, this study is the first to report the ChEs inhibitory activity of coumestrol, derived via enzyme kinetic analysis and molecular docking simulation. Furthermore, the identification of coumestrol as a dual ChE and BACE1 inhibitor could set a benchmark for treating AD. Puerarol exhibited hydrophobic interactions with Asp32 and Asp228, major catalytic residues. On the other hand, it interacted with Thr232, an important allosteric residue, specifically through a hydrogen bonding interaction. Thus, puerarol exhibited inhibitory activity against the target protein in terms of binding efficiency. In particular, this study is the first to report the BACE1 inhibitory activity of puerarol derived via enzyme kinetic analysis and molecular docking simulation.

## 4. Materials and Methods

### 4.1. Chemicals and Reagents

Electric eel AChE (EC 3.1.1.7), horse serum BChE (EC 3.1.1.8), acetylthiocholine iodide (ATCh), butyrylthiocholine chloride (BTCh), 5,5-dithiobis[2-nitrobenzoic acid] (DTNB) and berberine chloride were purchased from Sigma-Aldrich Co. (St. Louis, MO, USA). All required reagent-grade chemicals used in this study were purchased from commercial sources. Coumestrol and puerarol were isolated from *P. lobata* as described by Seong et al. [23]. The structures of coumestrol and puerarol are given in Figure 1.

### 4.2. DPPH Radical Scavenging Activity

DPPH radical scavenging activity was evaluated using the method of Blois [36], with slight modification. Test samples and DPPH were dissolved in methanol. Each test sample (160 µL) at various concentrations was added to 40 µL of DPPH solution (1.5 × 10^−4^ M). After gently mixing and standing at room temperature for 30 min, the optical density of the reactant was measured at 520 nm using a VERSAmax microplate spectrophotometer (Molecular Devices, Sunnyvale, CA, USA). The antioxidant activity of samples is expressed in terms of EC_50_ values (μM required to scavenge DPPH radical by 50%), which was calculated from the log-dose inhibition curve. *L*-Ascorbic acid was used as a positive control.

### 4.3. Assay of ONOO^−^ Scavenging Activity

ONOO^−^ scavenging activity was assessed using a modified Kooy’s method that involves the monitoring of highly fluorescent rhodamine 123, which is rapidly produced from non-fluorescent dihydrorhodamine (DHR) 123 in the presence of ONOO^−^ [37]. In brief, the rhodamine buffer (pH 7.4) consisted of 50 mM sodium phosphate dibasic, 50 mM sodium phosphate monobasic, 90 mM sodium chloride, 5.0 mM potassium chloride, and 100 μM diethylenetriamine pentaacetate. The final DHR 123 concentration was 5 μM. The assay buffer was prepared prior to use and placed on ice. Test samples were dissolved in 10% DMSO (100 μM final concentration). The background and final fluorescent intensities were measured 5 min after treatment with and without the addition of authentic ONOO^−^ (10 μM), dissolved in 0.3 N sodium hydroxide. The fluorescence intensity of oxidized DHR 123 was evaluated using a microplate spectrofluorometer (Molecular Devices, Sunnyvale, CA, USA) at excitation and emission wavelengths of 480 and 530 nm, respectively. Values of ONOO^−^ scavenging activity were calculated as the final fluorescence intensity minus the background fluorescence via the detection of DHR 123 oxidation. *L*-penicillamine was used as a positive control.

### 4.4. Western Blot Analysis for Inhibition of ONOO^−^-Mediated Nitrotyrosine Formation

The inhibition of ONOO^−^-nitrotyrosine formation was evaluated using the method of Aulak et al. [38]. Samples were added to BSA and mixed with ONOO^−^ (200 µM). After a 10 min incubation period (room temperature), the sample solution was added to Bio-Rad 2X Laemmli Sample buffer with mercaptoethanol and then boiled for 7 min. The reactant was resolved in 10% polyacrylamide gel via electrophoresis and transferred to PVDF membranes. Pre-stained blue protein markers were used for molecular weight determination. Monoclonal anti-nitrotyrosine antibody (85 kDa) was used as a primary antibody and horseradish peroxidase-conjugated sheep anti-mouse secondary antibody was used as a secondary antibody. Antibody labeling was visualized using the Supersignal West Pico Chemiluminescent substrate (Pierce Chemical Co., Rockford, IL, USA). Densitometric analysis of the western blot results was determined using a CS analyzer v3.00 (ATTO Corp.).

### 4.5. In Vitro ChE Enzyme Assay

The inhibitory activity of coumestrol and puerarol against ChEs was measured using the spectrophotometric methods reported by Ellman et al. [39] and Jung et al. [40]. ATCh and BTCh were used as substrates to assay the inhibition of AChE and BChE, respectively. All reactions were performed in triplicate and were recorded in a 96-well microplate format using a microplate spectrophotometer (Molecular Devices, Sunnyvale, CA, USA). Percent inhibition was calculated as (1 − S/E) × 100, where E and S were the enzyme activities without and with the test sample, respectively. ChE inhibitory activities were expressed in terms of IC_50_ value (μM required to inhibit hydrolysis of the substrate, ATCh or BTCh, by 50%) as calculated from the log-dose inhibition curve. Berberine was used as the positive control [41] The tested concentration range was 5–200 μM for coumestrol and puerarol, and 0.1–55 μM for berberine.

### 4.6. In Vitro BACE1 Enzyme Assay

The in vitro BACE1 enzyme assay was carried out according to the manufacturer’s recommended protocol with minor modifications. Briefly, a mixture of 10 mL of assay buffer (50 mM sodium acetate, pH 4.5), 10 mL of BACE1 (1.0 U/mL), 10 mL of substrate (750 nM Rh-EVNLDAEFK-Quencher in 50 mM, ammonium bicarbonate) and 10 mL of the test sample was dissolved in 10% DMSO and incubated for 60 min at 25 °C in the dark. Proteolysis of the two fluorophores (Rh-EVNLDAEFK-Quencher) by BACE1 was determined by monitoring formation of the fluorescent donor (Rh-EVNL) at wavelengths of 545 nm for excitation and 585 nm for emission. Fluorescence was measured with a microplate spectrofluorometer (Gemini EM, Sunnyvale, CA, USA). Quercetin was used as a positive control [42].

### 4.7. Kinetic Parameters for AChE, BChE, and BACE1 Inhibition

To determine the mechanism of inhibition, AChE, BChE, and BACE1 inhibition was evaluated by monitoring the effects of different concentrations of substrate (0.4 to 0.8 mM for AChE and BChE; 250 to 750 nM for BACE1). Specifically, AChE, BChE, and BACE1 inhibition assays were performed as described above, but the substrate concentration was varied. Inhibition modes of coumestrol and puerarol against ChEs and BACE1 were determined by Lineweaver–Burk plots [43]. Inhibition constants (*K_i_*) were determined by secondary plots [22].

### 4.8. ChEs and BACE1 Molecular Docking Simulations

To simulate the structure of the enzyme–inhibitor complex and to ensure accuracy, repeatability, and reliability of the docking results, we employed AutoDock 4.2 software. In our study, coumestrol was tested for ChEs inhibition, while puerarol was tested for BACE1 inhibition. For docking studies, the crystal structures of the AChE [44], BChE [45], and BACE1 [46] protein targets were obtained from the protein sequence alignment (Protein Data Bank (PDB ID: 1ACJ, 4BDS, and 2WJO, respectively)). The 3D structure of coumestrol, puerarol, donepezil, cryptotanshinone, and 5,7,4′-trimethoxyflavone (TMF) was obtained from the Pubchem Compound Database (NCBI), with compound CIDs of 5281707, 44257531, 3152, 160254, and 79730, respectively. Automated docking simulation was performed with AutoDock 4.2 using AutoDock Tools to assess the appropriate binding orientations and conformations of the ligand molecules with different protein inhibitors [47,48]. For docking calculations, grid maps were generated with the Autogrid program where the grid box size of 60 × 60 × 60 Å centered on the binding site of reported catatlytic and allosteric inhibitors. The docking protocol for rigid and flexible ligand docking consisted of 10 independent genetic algorithms, and the other parameters used were ADT defaults. The reported allosteric inhibitors, donepezil [49], cryptotanshinone [50], and TMF [51] were used to compare the interaction residues and aspect. The results were visualized and analyzed using PyMOL (v1.7.4, Schrödinger, LLC, Cambridge, MA, USA) and LigPlot^+^ (v1.4.5, European Bioinformatics Institute, London, UK).

### 4.9. Statistical Analysis

ANOVA and Student’s *t*-test were used to analyze statistics. Data are presented as the mean ± SEM of at least four independent experiments.

## Figures and Tables

**Table 1:** 2,2-Diphenyl-1-picrylhydrazyl (DPPH) and peroxynitrite scavenging activity of coumestrol and puerarol.

Samples	EC_50_ (μM) ^a^	
	DPPH	Peroxynitrite
Coumestrol	53.98 ± 1.00	1.17 ± 0.11
Puerarol	82.55 ± 1.33	6.99 ± 0.30
*L*-Ascorbic acid ^b^	17.37 ± 0.32	‒
*L*-Penicillamine ^b^	‒	6.90 ± 0.08

^a^ The 50% inhibitory concentration (EC_50_) values (μM) were calculated from a log dose inhibition curve and expressed as mean ± S.E.M of triplicate experiments; ^b^ Positive controls. (‒) no test.

**Table 2:** Cholinesterases and BACE1 inhibitory activity and enzyme kinetic analysis of coumestrol and puerarol.

Samples	IC_50_ (µM) ^a^			Inhibition Modes	*K_i_* Values ^b^	
	AChE	BChE	BACE1		*K_ic_*	*K_iu_*
Coumestrol	42.33 ± 1.29	24.64 ± 2.28	51.04 ± 1.86	Competitive ^c^ Mixed-type ^d^	48.91 12.07	‒ 41.87
Puerarol	144.80 ± 2.46	>200	28.17 ± 2.48	Mixed-type ^e^	33.8	73.19
Berberine ^f^	2.22 ± 0.02	12.30 ± 1.15	‒	‒	‒	‒
Quercetin ^f^	‒	‒	21.28 ± 1.42	‒	‒	‒

^a^ The 50% inhibitory concentration (EC_50_) values (μM) were calculated from a log dose inhibition curve and expressed as mean ± S.E.M of triplicate experiments; ^b^ Determined using secondary plot. *K_ic_* (binding constants of inhibitor with free enzyme) values were determined by secondary plots of the *K*_mapp_/*V*_maxapp_ of inhibitor concentrations. *K_iu_* (binding constants of inhibitor with enzyme-substrate complex) values were determined by secondary plots of the 1/*V*_maxapp_ of inhibitor concentrations; ^c,d^ Enzyme kinetic analysis with ^c^ AChE and ^d^ BChE were determined using Lineweaver-Burk plots; ^e^ Enzyme kinetic analysis with BACE1 were determined using Lineweaver-Burk plots; ^f^ Positive controls. (‒) no test.

**Table 3:** Molecular interaction of cholinesterases with coumestrol as well as reported inhibitors.

Compounds	Binding Score (kcal/mol)	H-Bonds Interacting Residues (No. of H-bond)	Hydrophobic Interacting Residues
**AChE (1ACJ)**			
Coumestrol	−8.63	Glu199 (1)	Trp84, Gly117, Tyr130, Phe330, Tyr334, Trp432, Met436, Ile439, His440, Gly441, Tyr442
Tacrine ^a^ (Catalytic inhibitor)	−9.80	His440 (1)	Tyr442, Phe330, Trp84, Gly118, Trp432, Gly441, Tyr334, Glu199
Donepezil ^a^ (Allosteric inhibitor)	−10.6	‒	Tyr70, Ile275, Asp276, Trp279, Ile287, Phe288, Arg289, Tyr334, Tyr121, Ser286, Phe290, Phe330, Phe331
**BChE (4BDS)**			
Coumestrol (Catalytic inhibition mode)	−8.28	Glu197 (1), Ser198 (1), Leu286 (1), His438 (1)	Trp82, Gly116, Gly117, Trp231, Phe398, Ile442
Coumestrol (Allosteric inhibition mode)	−8.67	Asp70 (1), Glu197 (1)	Gln67, Ile69, Trp82, Asn83, Gly115, Thr120, Gly121, Tyr128, Gly439, Try440
Tacrine ^a^ (Catalytic inhibitor)	−8.60	His438 (1)	Tyr332, Trp430, Trp82, Ala328, Glu197
Cryptotanshinone ^a^ (Allosteric inhibitor)	−7.80	‒	Asp70, Try82, Ala328, Tyr332, Trp430, Tyr440

^a^ Used as positive controls.

**Table 4:** Molecular interaction of BACE1 (2WJO) active site with puerarol as well as reported inhibitor QUD and TMF.

Compounds	Binding Score (kcal/mol)	H-Bonds Interacting Residues (No. of H-bond)	Hydrophobic Interacting Residues
Puerarol (Catalytic inhibition mode)	−8.80	Thr231 (1)	Asp32, Gly34, Val69, Tyr71, Trp76, Phe108, Trp115, Ile118, Asp228
Puerarol (Allosteric inhibition mode)	−8.03	Thr232 (1)	Ser10, Gly156, Ala157, Trp277, Gln303, Gln304, Arg307, Pro308, Asp318, Tyr320, Ala335, Val336, Gln339, Val361,
QUD ^a^ (Catalytic inhibitor)	−9.30	Asp228 (1), Asp32 (2), Gly230 (1)	Lys107, Lys75, Gly74, Leu30, Thr231, Val69, Tyr198, Ile226, Thr329, Gly34, Arg235, Ser35, Tyr71, Ile118
TMF ^a,b^ (Allosteric inhibitor)	−7.80	Gly11 (1)	Ser10, Tyr14, Thr232, Trp277, Glu303, Gln304, Leu306, Arg307, Pro308, Tyr320, Ala335, Val336, Glu339

^a^ Used as positive controls; ^b^ 5,7,4′-Trimethoxyflavone.
